# Prospective Analysis of Clinicopathologic Correlates of At-Home Feline Infectious Peritonitis Treatment Using GS-441524

**DOI:** 10.3390/pathogens14050507

**Published:** 2025-05-21

**Authors:** Kelly Larson, Emma Hart, Rosa Negash, Wendy Novicoff, Nicole Jacque, Samantha Evans

**Affiliations:** 1 Department of Microbiology, Immunology, and Pathology, Colorado State University, Ft. Collins, CO 80521, USA; 2 Department of Veterinary Biosciences, The Ohio State University, Columbus, OH 43210, USA; hart.1105@buckeyemail.osu.edu (E.H.); negash.13@buckeyemail.osu.edu (R.N.); 3 Department of Orthopedic Surgery & Public Health Sciences, University of Virginia, Charlottesville, VA 22903, USA; wnovicoff@gmail.com; 4 Independent Researcher, San Jose, CA 95123, USA; nicole@fipglobalcats.com

**Keywords:** Feline Infectious Peritonitis (FIP), complete blood count (CBC), biochemistry, prognostic indicators, GS-441524, antiviral

## Abstract

Feline Infectious Peritonitis (FIP) is caused by a systemic feline coronavirus (FCoV). Prior to June 2024, compounded FIP treatment was unavailable for prescription by veterinarians in the United States, leading to many cat owners obtaining treatment through unlicensed “black market” sources. We hypothesized that clinicopathologic data could provide insight on prognostic indicators for the treatment of FIP with GS-441524. This study used data gathered via surveys from 126 cat owners who used “black market” GS-441524 for their cats. We compared bloodwork parameters over twelve weeks of treatment. None of the clinicopathologic correlates, when analyzed via two-sample *t*-tests, produced statistically significant results between cured, deceased, and relapsed groups. Within cats considered cured, it was observed that hematocrit (HCT) and white blood cell (WBC) values were within normal limits by the 2–6-week period. Cats who died during the study had lower HCT and higher WBC values within the 2–6-week period. Trends were also seen in A/G and total bilirubin (T-BIL), with deceased patients showing a higher A/G ratio and lower value than those in the cured group. Overall, these data demonstrate a lack of traditional clinicopathologic parameters which are consistently predictive of FIP therapy success. Other predictors of outcome with antiviral therapy should be pursued.

## 1. Introduction

FIP is a feline disease caused by a systemic coronavirus that arises from certain strains of FCoV [1]. For many years, FIP had no proven effective treatments until antiviral candidates such as GS-441524 were identified and then used starting around 2019–2020 [2]. Since 2016, antiviral drugs, such as the protease inhibitor GC376 and nucleoside analog GS-441524, the active form of the prodrug Remdesivir (GS-5734), that effectively inhibit the replication of FCoV have been developed [3]. Both Remdesivir and GS-441524 have been demonstrated to be efficacious in suppressing FIPV replication in in vitro studies [4]. Prior to June 2024, compounded FIP treatment, like GS-441524, was unavailable for prescription through United States veterinarians. Despite this, the uniformly fatal nature of the disease, as well as information on social media about the efficacy of treatment using GS-441524, led many cat owners to seek black market sources for treatment. Indeed, several studies have since confirmed GS-441524 as a highly effective treatment, noting survival rates of 81% or higher [2,3,4,5,6].

Within the current research on the topic of efficacy, treatment guidelines, and prognostic indicators of the treatment of FIP with nucleoside analogues, like GS-441524, there remain significant gaps in our knowledge. We hypothesize that by analyzing clinicopathologic data from cats diagnosed with FIP being treated with “black market” GS-441524 at home, we could provide insights on prognostic indicators for therapeutic success. Due to the unlicensed nature of the drug procured by pet owners, we cannot confirm the manufacturers or their country of origin. However, we have tested 127 different unlicensed products and found them all to contain GS-441524 [7]. Currently, treatment guidelines advise daily treatment with GS-441524 for a minimum of twelve weeks, as that is when clinical signs and biochemical abnormalities are shown to have been resolved [8]. Daily treatment with GS-441524 using a recommended 2–5 mg/kg subcutaneously demonstrated effective blood levels over a 24 h during pharmacokinetic studies in laboratory cats [9,10]. However, recent evidence suggests that shorter treatment times may be sufficient in some cases [7]. With the frequently prohibitive cost of this treatment and difficulty of long-term administration, a better understanding of therapeutic correlates and predictors of positive response would be of benefit. This study aims to provide insight on patterns within the clinicopathologic correlates of therapy to inform future treatment guidelines and research on FIP.

## 2. Materials and Methods

### 2.1. Study Design and Protocol

Participants using unlicensed GS-441524 to treat their cats with suspected FIP were recruited from online social media groups in 2022, primarily Meta’s Facebook platform with users predominantly from the United States. With institutional review board approval (Ohio State University protocol #2021E0162), six surveys were designed, using Qualtrics XM software (version January 2022, Qualtrics, Provo, UT, USA) to capture information covering efficacy, survival rate, dosing, treatment completion, complications, relapsing, and veterinary support [11]. Participants were recruited via a digital study flyer with a link to a pre-enrollment survey, word of mouth, and social media posts in groups such as FIP Warriors [11]. No monetary incentive was given, and all participants were recruited at diagnosis or the initiation of therapy for their pet(s) to prevent bias toward positive outcomes [11]. Surveys were sent to participants weekly to gather information about diagnosis, treatment, and outcome for a minimum of twelve weeks. The complete blood count (CBC) and biochemistry panels (laboratory data) analyzed in this study originated from uploaded flies from surveys completed by cat owners who were treating FIP at-home with “black market” GS-441524. A report on owner experience and veterinary involvement with unlicensed GS-441524 treatment in this same cohort was previously published by our group [11].

### 2.2. Inclusion Criteria

For the purpose of this study, “cured” is defined as alive at the end of the study with the resolution of biochemical abnormalities as reported by participants’ weekly surveys. “Deceased” is defined as death (including euthanasia) having occurred at any point during the study as reported by participants’ weekly surveys. “Relapsed” is defined as a patient who was reported cured by participants’ weekly surveys then reported to have deteriorated with the presence of abnormal biochemical data without a known outcome of cured or deceased. Generally, data points were groups by outcome (cured, deceased, or relapsed) to compare and find patterns in biochemical data across shared outcomes. Within each known outcome (cured, deceased, or relapsed), data were sorted by biochemical value from laboratory results to compare and identify patterns.

To be included in this data analysis, cured patients needed at least two different dates of laboratory results, to be able to compare values. Deceased and relapsed cats who had any number of laboratory result data points provided were also included, due to their smaller sample size within this study. After a year of open enrollment, 579 individuals responded to the initial interest form survey, and 383 individuals continued and completed the enrollment survey [11]. Of the 383 individuals who continued with the surveys, 126 individual cats met the inclusion criteria, and their data were downloaded, cataloged, and analyzed.

### 2.3. Statistical Analysis

Laboratory results attached to weekly surveys were manually input into an excel spreadsheet. Colorado State University (CSU) reference intervals (RIs) for routine hematologic and biochemical parameters are the following: hemoglobin (HGB) 9.8–15.5 g/dL, HCT 32–47%, red blood cell (RBC) 6.5–10 10^6^/uL, mean corpuscular value (MCV) 40–52 fL, red cell distribution width (RDW) 13.5–19%, mean corpuscular hemoglobin concentration (MCHC) 32–36 mg/dL, WBC 4.0–14.0 10^3^/uL, reticulocyte 0–50 10^3^/uL, platelet (PLT) 200–500 10^3^/uL, mean platelet volume (MPV) 10–22.5 fL, neutrophil 2–12 K/uL, lymphocyte 1.5–6 K/uL, monocyte 0–0.8 K/uL, eosinophil 0–1.2 K/uL, basophil 0–0.1 K/uL, glucose 68–140 mg/dL, creatinine (CREAT) 0.8–2.4 mg/dL, BUN 18–35 mg/dL, phosphorus 3–6 mg/dL, calcium (Ca) 9.2–11.1 mg/dL, total protein (TP) 6.3–8.0 g/dL, albumin 3.1–4.4 g/dL, globulin 2.7–4.2 g/dL, A/G ratio 0.8–1.6, cholesterol 95–270 mg/dL, T-BIL 0.0–0.1 mg/dL, alkaline phosphatase (ALP) 10–80 U/L, alanine aminotransferase (ALT) 30–140 U/L, gamma-glutamyl transferase (GGT) 0–0.5 U/L, sodium (Na) 149–157 mmol/L, potassium (K) 3.7–5.4 mmol/L, and chloride 115–125 mmol/L. These RIs were used in the graphical representation of these data. Statistical analysis was performed in GraphPad Prism version 10.1.2 for Windows (GraphPad Software, Boston, MA, USA, www.graphpad.com, accessed on 26 August 2024). Due to the small sample size of cats in the relapsed and deceased groups, these groups were combined into one for comparison with cured cats. Two-sample *t*-tests were used for comparisons between these two groups.

## 3. Results

Laboratory data came from a variety of sources (IDEXX, Antech, etc.) and were noted alongside the data input. Notably, no lab data were collected from CSU labs. RIs from CSU were used for visual representation only. Of the 169 individual cats surveyed, 126 met the inclusion criteria: 105 cured, 17 deceased, and 4 relapsed cats fit the inclusion criteria. Of the total patients surveyed and treated with GS-441524, 83.3% were cured, 13.5% were deceased, and 3.2% were relapsed. Of the 169 cats, 18 were intact males, 98 were neutered males, 9 were intact females, and 44 were spayed females. Additionally, 85 cats were reported to have wet, 30 had dry, 7 had neurological, 3 had ocular, and 40 had mixed (meaning two or more of the others) phenotypes of FIP. Given the variability in time points of the bloodwork collection, CBC and biochemistry data were grouped relative to the initiation of therapy (time 0) into the following categories: −2 to 0 weeks (diagnosis phase), 0–2 weeks, 2–6 weeks, 6–12 weeks, and 12–20 weeks post initiation of therapy.

None of the clinicopathologic parameters correlated, when analyzed via two-sample *t*-tests, produced statistically significant results between cured and the combination of deceased and relapsed groups. There were not enough data points within the deceased and relapsed groups to allow for analysis of parameters over time. This was in part due to not having data for each time category from each cat across parameters. Due to the limited relevant statistical analyses able to be performed on this data, we focused on the two-sample *t*-test performed for comparison between groups and possible patterns visible to the naked eye.

**Figure 1:**
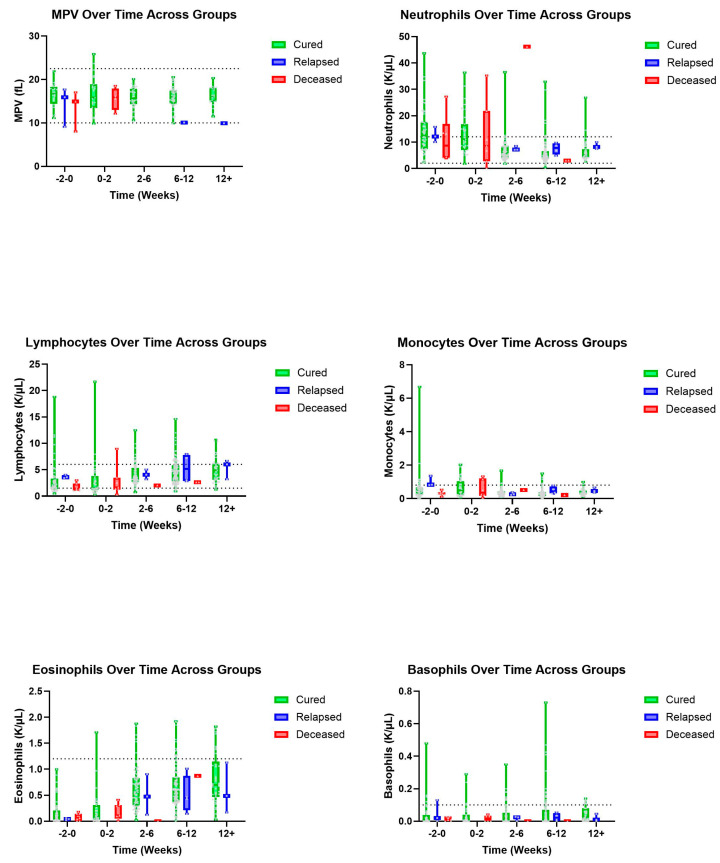
Additional leukocyte and PLT parameters (top–bottom and left–right): MPV, neutrophils, lymphocytes, monocytes, eosinophils, and basophils over time, in weeks, for cured, relapsed, and deceased patients. Dashed lines indicate CSU RIs. The grey dots represent individual data points.

**Figure 2:**
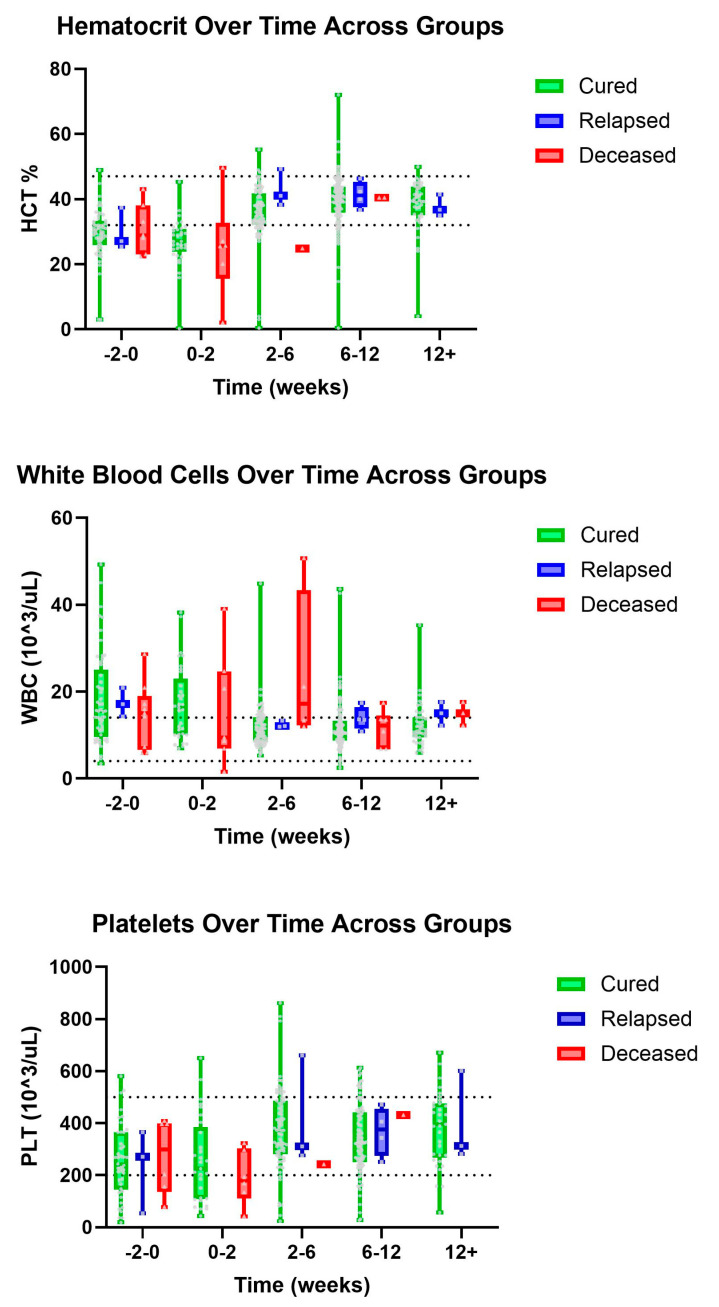
Major hematology parameters (top–bottom): HCT, WBC, and PLT over time, in weeks, for cured, relapsed, and deceased patients. Dashed lines indicate CSU RIs. The grey dots represent individual data points.

There was a visual trend within lymphocytes that cats considered cured had lymphocyte values within normal limits from the initiation of treatment with a gradual increase over time, while cats within the deceased group had, on average, lower lymphocyte values both initially and then over time, despite a slight visual increase in lymphocyte count (Figure 1). Within the group of cats considered cured, it was observed that HCT values were within normal limits by the 2–6-week period, while cats who ultimately died during the study had lower HCT values within the 2–6-week period (Figure 2). Similarly, we observed that cured cats tended to have normal WBC values by the 2–6-week period, while cats from the deceased group had a higher average WBC value during the 2–6-week period (Figure 2).

**Figure 3:**
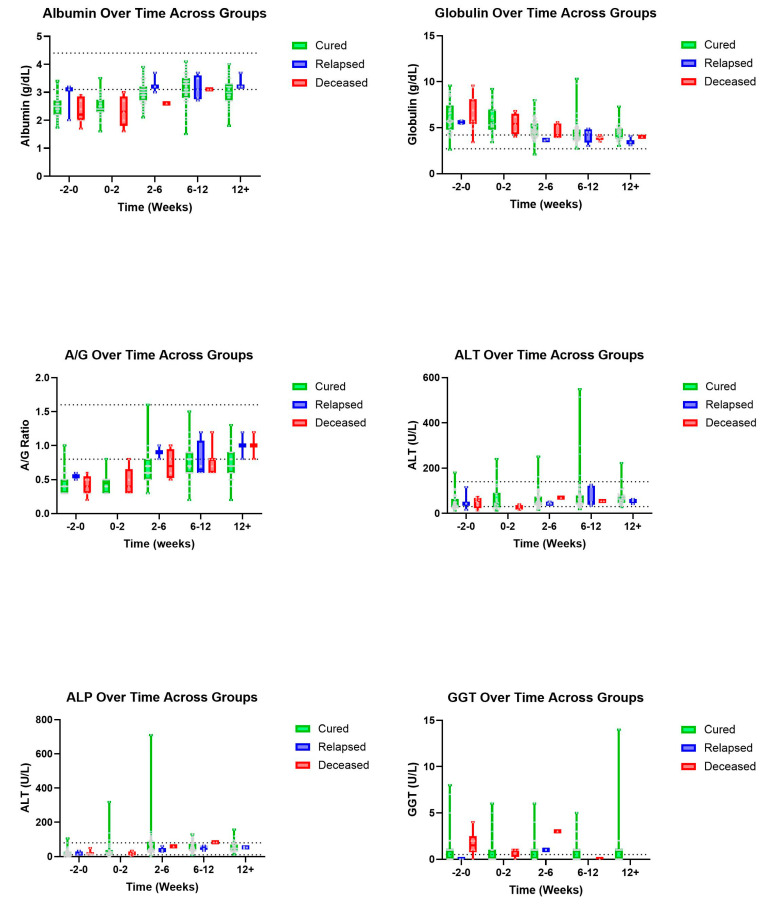
Additional biochemical parameters (top to bottom and left to right): Albumin, globulin, A/G, ALT, ALP, and GGT over time, in weeks, for cured, relapsed, and deceased patients. Dashed lines indicate CSU RIs. The grey dots represent individual data points.

**Figure 4:**
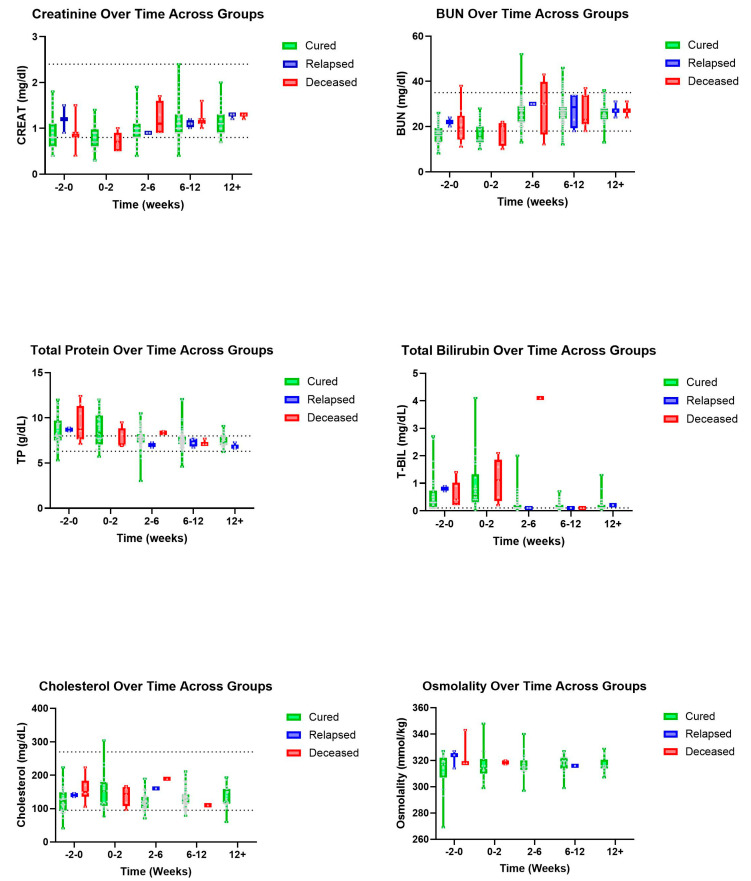
Additional biochemical parameters (top to bottom and left to right): CREAT, BUN, TP, T-BIL, cholesterol, and osmolality over time, in weeks, for cured, relapsed, and deceased patients. Dashed lines indicate CSU RIs. No CSU RI is available for osmolality. The grey dots represent individual data points.

**Figure 5:**
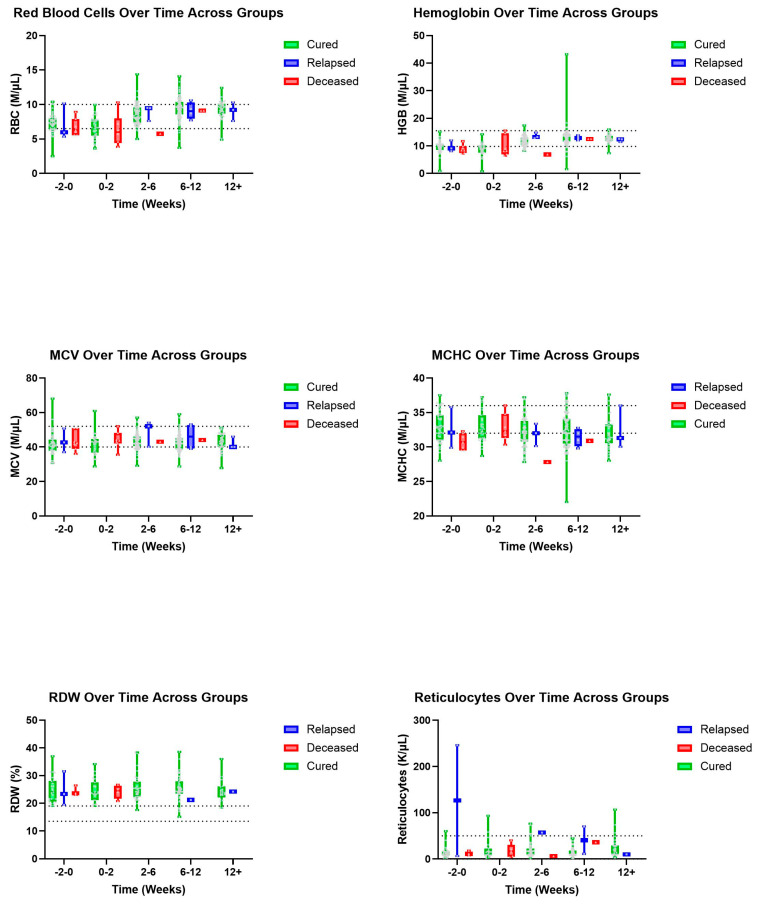
Additional erythrocyte parameters (top to bottom and left to right): RBC, HGB, HCV, HCHC, RDW, and reticulocytes over time, in weeks, for cured, relapsed, and deceased patients. Dashed lines indicate CSU RIs. The grey dots represent individual data points.

**Figure 6:**
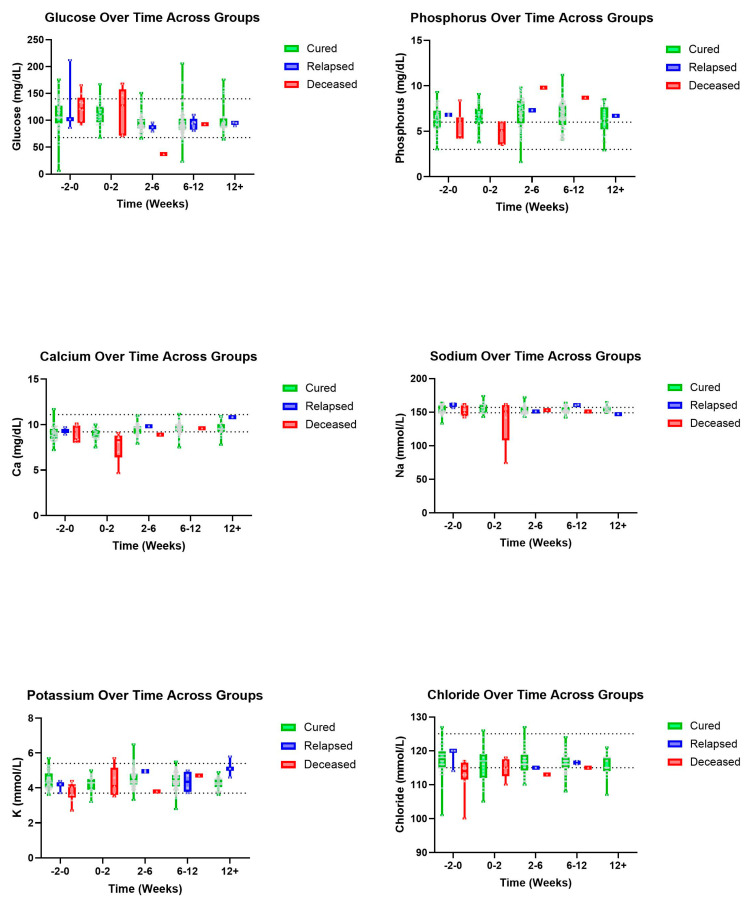
Biochemical parameters (top–bottom and left–right): Glucose, P, Ca, Na, K, and chloride over time, in weeks, for cured, relapsed, and deceased patients. Dashed lines indicate CSU RIs. The grey dots represent individual data points.

Trends were also seen in A/G ratios with deceased patients showing an, on average, higher ratio value than those in the cured group (Figure 3). A similar pattern was observed with T-BIL where deceased patients had a recorded higher, on average, T-BIL value than those in the cured group whose values showed within normal limits by 2–6 weeks (Figure 4). Other hematologic and biochemical values were recorded and analyzed, also producing no statistically significant findings (Figure 5 and Figure 6).

## 4. Discussion

Although there were some observable patterns within out data, they were found not to be statistically significant after an analysis of 126 individual cats’ laboratory data, collected via weekly, digital surveys, spanning their treatment for suspected FIP with unlicensed or “black market” GS-441524. Of note, our data display wide variation in values for many clinicopathologic parameters in cats that were ultimately categorized as cured. This supports that marked changes in routine laboratory data alone should not dissuade veterinarians or owners from attempting treatment of cats with suspected FIP.

One study reported that cats with an A/G ratio less than 0.5 were highly suspected of having FIP while those with an A/G ratio of greater than 0.8 had a very small chance of being diagnosed with FIP [12]. Within our study, 46.7% (49/105) of cats considered cured had A/G ratios greater than or equal to 0.8 reported at 6–12 and/or 12+ weeks. Additionally, 75% (3/4) of the relapsed cats reported A/G ratios of 0.8 or greater at 6–12 and/or 12+ weeks. Given the nature of this study, as well as the diagnostic challenge that FIP can present, it remains possible that some of the deceased patients died of non-FIP disease, which may explain why they trended toward higher A/G ratios. One study reported A/G ratios rising gradually from between week two of treatment and week nine during a twelve-week treatment plan of cats with FIP being treated with GS-441524 [2]. Another reported an A/G ratio having returned to the RI 6–7 weeks after starting antiviral treatment with GS-441524 [13]. Generally, our data support this trend with visual representations depicting a gradual increase in A/G ratios over the course of a similar twelve-week treatment for FIP with GS-441524. Although, our data did not show a consistent normalization of A/G ratios by the 2–6- nor the 6–12-week period. Taken together, the A/G ratio may not be a highly useful (sensitive or specific) test for monitoring the success of FIP therapy.

A previous study recorded that cats who are more likely to survive with treatment had a pretreatment T-BIL of below 0.5 mg/dL, while those who exceeded 4.0 mg/dL had a decreased survivability [14]. Hyperbilirubinemia has also been reported as resolved within two to three weeks of treatment with GS-441524 [2]. Our data also support this trend, with deceased cats having a mean T-BIL greater than cured cats at diagnosis and at the 0–2-week time point. Although these trends lacked statistical significance, T-BIL merits further study as a prognostic indicator for FIP treatment. Importantly, though, the range of T-BIL values in the cured group in our dataset was great (indeed, it extended beyond the range for the deceased group), demonstrating that treatment is not futile for cats with a T-BIL over 0.5 mg/dL.

Similarly, these results suggest that larger studies examining WBC, HCT, and lymphocytes as prognostic indicators may be beneficial, possibly using machine learning algorithms to analyze patterns across many parameters. Within our data, we observed that cured cats tended to have WBC values that normalized by the 2–6-week period, while cats from the deceased group had a higher average WBC value during the same time period.

Absolute lymphocyte count in blood was noted as the most accurate predictor of disease outcome, with 18 cats that died within 4 weeks of infection with FIP showing a profound drop in blood lymphocytes starting at 2 weeks [15]. While our data did not mirror this exactly, there was a visual trend wherein cats considered cured had lymphocyte values within normal limits from the initiation of treatment with a gradual increase over time. Comparatively, cats within the deceased group had slightly lower lymphocyte values both initially and over time.

Within our study it was observed that HCT values were within normal limits by the 2–6-week period for those cats considered cured, while cats who ultimately died during the study had lower HCT values within the same time period. This aligns with another study that reported HCT values returning to the RI 6–7 weeks after beginning treatment with GS-441524 [13]. Despite this study’s analysis for trends within HCT lacking statistical strength, we do have visualized patterns that support the use of HCT as a possible prognostic indicator for FIP. However, it is important to note that there is great overlap between groups for all of the parameters described.

It is interesting that our results were unable to identify clear prognostic indicators in this large group of cats, though a lack of clinicopathologic predictors of FIP therapy success is an important outcome. Other predictors of outcome with antiviral therapy are needed and should be pursued as a research direction for this field. Importantly, we were unable to evaluate any acute phase proteins as these parameters are almost never assessed by United States laboratories.

Due to the survey-based nature of this study, our results may have been impacted by reporting errors from lay owners, or by human error during the completion of surveys of the transfer of the uploaded data. Further, black market formulations of GS-441524 are largely from unknown sources/manufacturers and known to have significant variability in content/concentration, which could be a determinant of outcome that we were unable to account for here [7]. Nevertheless, the overall success rate (~83%) in this prospective survey study of black-market GS-441524 use for FIP was in line with clinical trial data using compounded formulations of GS-441524 at other institutions, suggesting that this variability may not have had a huge impact on overall therapeutic success. One such study reported an 81.3% remission rate for cats diagnosed with FIP and treated with GS-441524 [13]. Another limitation of this paper was that laboratory data collected came from multiple different lab sources. This means that our study could not account for different types of instrumentation, methodologies, and RIs used across these various entities. Despite these limitations, this study represents the largest prospective analysis of clinicopathologic laboratory data from cats treated with GS-441524 to date.

Regulated prescription treatment was released in the United States as of June 2024. Comparative studies from the “black market” treatment and the prescription treatment should be investigated as a means of measuring treatment quality and efficacy, while encouraging movement away from black market sources and toward veterinarian-led, regulated treatment. While compounded FIP treatment options make FIP a highly treatable disease, further research to refine treatment guidance and optimize protocols would be beneficial. Regulated treatment options greatly increase the veterinary community’s ability to study and collect data on treatment efficacy and prognostic indicators and will help to address many of the limitations impacting the present study. Overall, these data demonstrate a lack of traditional clinicopathologic parameters observable as consistently predictive of FIP therapy success, indicating that marked CBC or biochemistry abnormalities should not discourage treatment for FIP. Other predictors of outcome with antiviral therapy should be pursued in future research.

## Data Availability

All surveys and anonymized laboratory data are available upon request.

## Abbreviations

The following abbreviations are used in this manuscript:

FIP	Feline Infectious Peritonitis
FCoV	Feline coronavirus
CBC	Complete blood count
HCT	Hematocrit
WBC	White blood cell
PLT	Platelets
CREAT	Creatinine
TP	Total protein
T-BIL	Total bilirubin
A/G	Albumin/Globulin
HGB	Hemoglobin
RBC	Red blood cell
RDW	Red cell distribution width
MPV	Mean platelet volume
Ca	Calcium
ALP	Alkaline phosphatase
ALT	Alanine aminotransferase
GGT	Gamma-glutamyl transferase
Na	Sodium
K	Potassium
P	Phosphorus
CSU	Colorado State University
RIs	Reference intervals
